# Cynæcological Society of Boston

**Published:** 1869-02

**Authors:** 


CYNÆCOLOGICAL SOCIETY OF BOSTON.—This Society organize with the following preamble: "The undersigned, desirous of advancing the study of the Diseases of Women, hereby associate themselves together with that intent, and adopt for their government the appended constitution and by-laws." The constitution determines the *name, objects, who may be members and code of ethics.* The code of ethics is this: "The Society recognizes the code of ethics of the American Medical Association as binding upon its members." The by-laws regulate all other matters of the Society, and are too numerous for notice. Thus it will be seen that our neighbors are reducing the study of the diseases of women to a system, arranged with a *President*, a *Secretary* and a *Treasurer*.

